# Study of Ethylene-Removing Materials Based on Eco-Friendly Composites with Nano-TiO_2_

**DOI:** 10.3390/polym15163369

**Published:** 2023-08-11

**Authors:** Alba Maldonado, Paulina Cheuquepan, Sofía Gutiérrez, Nayareth Gallegos, Makarena Donoso, Carolin Hauser, Marina P. Arrieta, Alejandra Torres, Julio Bruna, Ximena Valenzuela, Abel Guarda, María Galotto, Francisco Rodríguez-Mercado

**Affiliations:** 1 Packaging Innovation Center (LABEN–Chile), Universidad de Santiago de Chile, Obispo Umaña 050, Santiago 9170201, Chile; paulina.cheuquepan@usach.cl (P.C.); sofia.gutierrez.b@usach.cl (S.G.); nayareth.gallegos@usach.cl (N.G.); makarena.donoso@usach.cl (M.D.); alejandra.torresm@usach.cl (A.T.); julio.bruna@usach.cl (J.B.); ximena.valenzuela@usach.cl (X.V.); abel.guarda@usach.cl (A.G.); maria.galotto@usach.cl (M.G.); 2 Center for the Development of Nanoscience and Nanotechnology (CEDENNA), Universidad de Santiago de Chile, Alameda 3363, Santiago 9170022, Chile; 3 Department of Applied Chemistry, Nuremberg Institute of Technology Georg Simon Ohm, Keßlerplatz 12, 90489 Nuremberg, Germany; carolin.hauser@th-nuernberg.de; 4 Departamento Ingeniería Química Industrial y del Medio Ambiente, Escuela Técnica Superior de Ingenieros Industriales, Universidad Politécnica de Madrid, (ETSII-UPM), Calle José Gutiérrez Abascal 2, 28006 Madrid, Spain; m.arrieta@upm.es; 5 Department of Food Science and Technology, Faculty of Technology, Universidad de Santiago de Chile, Avenida Víctor Jara 3769, Santiago 9170124, Chile

**Keywords:** titanium dioxide, photocatalytic activity, ethylene-removing, active packaging, nanocomposites, eco-friendly materials

## Abstract

Ethylene is a phytohormone that is responsible of fruit and vegetable ripening. TiO_2_ has been studied as a possible solution to slowing down unwanted ripening processes, due to its photocatalytic capacity which enables it to remove ethylene. Thus, the objective of this study was to develop nanocomposites based on two types of eco-friendly materials: Mater-Bi^®^ (MB) and poly(lactic acid) (PLA) combined with nano-TiO_2_ for ethylene removal and to determine their ethylene-removal capacity. First, a physical–chemical characterization of nano-TiO_2_ of different particle sizes (15, 21, 40 and 100 nm) was done through structural and morphological analysis (DRX, FTIR and TEM). Then, its photocatalytic activity and the ethylene-removal capacity were determined, evaluating the effects of time and the type of light irradiation. With respect to the analysis of TiO_2_ nanoparticles, the whole samples had an anatase structure. According to the photocatalytic activity, nanoparticles of 21 nm showed the highest activity against ethylene (~73%). The results also showed significant differences in ethylene-removal activity when comparing particle size and type and radiation time. Thus, 21 nm nano-TiO_2_ was used to produce nanocomposites through the melt-extrusion process to simulate industrial processing conditions. With respect to the nanocomposites’ ethylene-removing properties, there were significant differences between TiO_2_ concentrations, with samples with 5% of active showed the highest activity (~57%). The results obtained are promising and new studies are needed to focus on changes in material format and the evaluation in ethylene-sensitive fruits.

## 1. Introduction

Ethylene (C_2_H_4_) is an unsaturated hydrocarbon and a gaseous plant hormone that is involved in almost all phases of fruit and vegetable (F&V) growth and development [1]. Ethylene can regulate ripening and senescence of F&V at molecular, biochemical and physiological levels, due to ethylene-stimulated gene expression for the synthesis of enzymes that promote these processes [2]. Although it is not yet clear whether ethylene is responsible for the initiation of ripening in F&V, or is an accelerator of ripening, it has been demonstrated that its elimination from storage chambers causes a slowdown in ripening [3]. In addition, it is well-known that ripening is dependent on whether the fruit is climacteric or non-climacteric. On the other hand, the relationship between F&V and ethylene has a higher consequence with the worldwide problem of food being lost or wasted. In this regard, the FAO has reported that approximately 45% of fruits and vegetables (F&V) are lost or wasted worldwide [4]. Due to their high metabolic activity, F&V are highly perishable products and the application of different methods to control their ripening in postharvest storage is key to adequately preserving these types of food.

Different technologies to minimize the negative effects of ethylene have been studied. These include modified and controlled atmospheres and cool storage; however, the use of chemical products has shown the best effects on F&V shelf-life [5]. Therefore, photocatalysis is a mechanism that has received a lot of attention in both scientific and industrial fields. Titanium dioxide (TiO_2_) is among the most well-researched substances. This oxide has been widely studied because it is harmless to both the environment and humans, as well as having applications in various productive areas, such as pharmaceutical, cosmetic and packaging industries, as well as being used as a pigment in paints and coatings, as a photocatalysts, and as an antimicrobial [6]. It is also a biologically and chemically stable substance with low cost, low toxicity, large surface area and high photocatalytic activity [7]. TiO_2_ can produce photocatalytic oxidation of C_2_H_4_ under ultraviolet (UV) radiation. The oxidation process begins on the TiO_2_ surface where UV radiation produces reactive oxygen species (ROS) which oxidize C_2_H_4_ into carbon dioxide (CO_2_) and water (H_2_O) [8,9]. Considering this property, TiO_2_ can be used as an active substance in the development of ethylene-removal plastic materials oriented to F&V. Moreover, application of titanium dioxide nanoparticles (nano-TiO_2_) has shown interesting results in ethylene control [10]. 

Considering the environmental problems generated by the inappropriate management of plastic materials from fossil resources, the generation of new eco-friendly plastic materials with specific functionality becomes relevant for industry and society. In this field, the use of eco-friendly polymers has been widely studied and it is possible to find several commercial alternatives that are available for the development of active materials. Poly(lactic acid) (PLA) and Mater-Bi^®^ are bioplastics which are produced from biological resources and are recognized for being biodegradable and compostable [11,12]. Due to its properties, PLA is recognized as the most suitable bio-based polymer material for food-packaging applications [13]. Indeed, it is versatile, compostable, recyclable, and moreover, it has high transparency, high molecular weight, high water-solubility resistance and good processability [14]. On the other hand, Mater-Bi^®^ is a commercial biodegradable material based on modified starch and bio-based polyester blends. Mater-Bi^®^ has important commercial applications because it shows interesting mechanical properties, thermal stability, processability and biodegradability [11]. 

Although, to date, it has been possible to find some studies where the antimicrobial and ethylene-removing activity of TiO_2_-based eco-friendly materials is reported [3,15,16], there have been few studies where this type of material was obtained through an extrusion process. In this sense, the aim of this study was to develop nanocomposites based on Mater-Bi^®^ and PLA with nano-TiO_2_ for ethylene removal and to evaluate the photocatalytic activity of different sizes of nano-TiO_2_ in removing ethylene. We used a melt-extrusion process which is potentially scalable to the industrial sector, for the development of active packaging for fruit and vegetables.

## 2. Materials and Methods

### 2.1. Materials

Nanoparticles of titanium dioxide (nano-TiO_2_) anatase were supplied by Sigma Aldrich (21 nm, 99.5%) and US Research Nanomaterials Inc. (Houston, TX, USA) (15 nm—99.5%, 40 nm—99.5% and 100 nm 99.9%). Its crystalline structure was anatase, 99.5% purity, with four different particle sizes: 15, 21, 40 and 100 nm. To determine photocatalytic activity methylene blue (MeB) (Sigma Aldrich, Darmstadt, Germany) was used. To elaborate nanocomposites, Mater-Bi^®^ EF51L (Novamont SpA, Milan, Italy) and PLA Ingeo Biopolymer 2003D (NatureWork, Plymouth, MN, USA) were provided used by Novamont SpA and NatureWork, respectively.

### 2.2. X-ray Diffraction (XRD)

To analyze the crystalline structure of nanoparticles, an analysis of X-ray diffraction was carried out on a powder X-ray diffractometer (Bruker, D8-Advance, Urbana, IL, USA) using CuKα (*λ* = 1.5406 Å) radiation at room temperature at 40 kV and 35 mA. All scans were performed at 2ϴ angles from 2° to 80° at 0.02° every 0.2 s. The lattice parameters were calculated according to Equation (1), which is used for tetragonal crystallites:

(1) $$\frac{1}{d^{2}}=\frac{h^{2}+k^{2}}{a^{2}}+\frac{l^{2}}{c^{2}}$$

where *h*, *k* and *l* are the Miller indices obtained from the crystallographic parameters of anatase (JCPDS 84-1286); *a* and *c* are the lattice parameters, and *d* value was obtained by the Bragg’s law Equation (2):

(2) nλ=2dsinθ

where *λ* is wavelength of incidence X ray (1.5406 Å), *θ* is peak position in radians, *n* is the order of diffraction (1) and *d* is the interplanar spacing or *d*-spacing.

### 2.3. Fourier Transform Spectroscopy (FTIR)

Spectroscopic analysis was carried out in an IR-ATR spectrometer (Bruker, Alpha, Germany). Absorbance was measured with a wavelength range from 4000 cm^−1^ to 400 cm^−1^ using 24 scans, and a resolution of 2 cm^−1^.

### 2.4. Transmission Electron Microscopy (TEM) and Scanning Electron Microscopy (SEM)

For nanoparticle samples, TEM analysis was performed on a transmission electron microscope (Hitachi, HT-7700, Tokyo, Japan) with a magnification of 50×. Before this analysis, the samples were diluted and dispersed in ethanol (95%) and placed on a copper grid. For SEM analysis the samples were coated with a gold–palladium film on a Hummer 6.2 metallizer. Images were obtained on a microscope (Tescan, Vega, Brno, Czech Republic) from 3 to 20 kV.

The proper incorporation and dispersion of nano-TiO_2_ into the polymeric matrix were evaluated by TEM. The nanocomposite was embedded in a low-viscosity TEM-grade epoxy resin (Taab Laboratories, Aldermaston, UK) and cured for 3 days at 40 °C. To observe the cross-section of the nanocomposite, the resins embedded with the films were cut with a Reichert Ultracut S Leica ultra-microtome (Leica Biosystems, Wetzlar, Germany) mounted on copper grids and the TEM images were obtained with a JEOL JEM-1010 transmission electron microscope. Only the samples of PLA could be measured because the samples of nanocomposites of MB could not be prepared by the method described above due to the soft composition of the MB matrix. The TiO_2_ particles’ sizes within the polymeric matrix were measured through ImageJ software (version 1.51 k, Java 1.6.0_24, Wayen Rasband, US National Institutes of Health, Bethesda, MD, USA).

### 2.5. Particle Sizes (PS)

The particle size of different nanoparticles was determined by dynamic light scattering (DLS) at 90° using a Zetasizer (NanoS90, Malvern Instruments, Worcestershire, UK). A refractive index of TiO_2_ equal to 2.49 and of continuous phase (ethanol) equal to 1.36 was used for measurements. The reported values corresponded to an average of 10 measurements, where each sample was measured in triplicate. 

### 2.6. Thermogravimetric Analysis (TGA)

Thermograms were obtained with a TGA/DSC 1 analyzer (Mettler Toledo, STARe System, Bern, Switzerland) with STARe V.12.0 software (Bern, Switzerland). Approximately 10 mg of sample was placed into an alumina crucible (70 µL). The experiment was performed in a high-purity dynamic nitrogen flow of 50 mL/min at a heating rate of 10 °C/min. The analysis was performed in a temperature range of 25 to 900 °C.

### 2.7. Photocatalytic Activity of TiO_2_

The photocatalytic activity of nano-TiO_2_ was determined against the degradation of MeB. This experiment considered the application of ultraviolet (UV) and visible (VIS) light. For this, irradiation chambers were used for UV light (Philips Actinic BL TL-D 15W/10, 365 nm, Hamburg, Germany) and VIS light (General Electric R7s 150 W/118 mm, 630 nm, CA, USA). For this purpose, 100 mL of a solution of MeB (17 ppm) was prepared, and 100 mg of TiO_2_ was added to this solution. The mixture was stirred in the dark for 30 min. The solution was then placed inside UV or VIS light chambers for 270 min, and 2 mL aliquots were taken every 30 min. The aliquots were centrifuged at 7000 rpm for 5 min in a centrifuge (Hettich, Universal 32R, Tuttlingen, Germany). Then, the supernatant was taken and placed into a cuvette (2 mL) and the absorbance was measured at 665 nm in a UV-VIS spectrophotometer (Shimadzu, UV-1900i, Tokyo, Japan). A calibration curve was prepared to determine the MeB concentration, where the absorbance of different MeB solutions was measured (1–20 ppm).

### 2.8. Ethylene-Removal Study

The study of the ethylene-removal kinetics of nano-TiO_2_ and nanocomposites was carried out according to the methodology described below. First, 0.15 g of TiO_2_ (1 g for nanocomposite samples) was introduced into a 22 mL vial; then, a gaseous ethylene–nitrogen mixture was injected into each of the vials at a concentration of 50,000 ppm. The vials were placed 15 cm from the lamp and were irradiated inside a special chamber with UV or VIS light, for 5 or 15 min at 20 °C. Then, samples from the head space were analyzed by gas chromatography at 0, 1, 2, 3, 4 and 5 days. For this, ethylene quantification was done in a Perkin Elmer Clarus 580 gas chromatograph (Waltham, MA, USA) with a Head Space Turbo Matrix 40-Perkin Elmer sampler (Waltham, MA, USA) with an RtTM-alumina PLOT column of 50 m length and 0.53 mm diameter. The gas chromatograph conditions consisted of an injector temperature of 180 °C, oven temperature of 180 °C and flame ionization detector (FID) temperature of 250 °C. Head space autosampler conditions included thermostatization temperature of 45 °C and needle and transfer-line temperature of 70 °C; N_2_ was used as carrier gas at a flow rate of 10 mL min^−1^.

### 2.9. Fabrication of Nanocomposites

Poly(lactic acid) (PLA) and Mater-Bi^®^ (MB) were used as polymeric matrices to produce nanocomposites with different contents of nano-TiO_2_ (0, 5 and 10% wt.). The nanocomposites were produced by extrusion, using a twin-screw micro-extruder (Thermo Scientific, Thermo Process 11, Erlangen, Germany). The extrusion conditions included a screw feed speed of 15 rpm, a twin-screw speed of 30 rpm, a die temperature of 190 °C and temperature profiles of 120–180 °C and 160–185 °C for PLA and MB, respectively.

### 2.10. Colorimetric Properties

Color analysis was carried out on an UltraScan VIS spectrophotometer (HunterLab, VA, USA). Prior to the measurement, the instrument was calibrated with the black and white cells. For this purpose, the sample was placed in a glass optics cell and inserted into the instrument, and the L*, a*, b* and ∆E_00_ were measured. In addition, ΔE parameter was determined from [17].

## 3. Results and Discussion

### 3.1. Characterization of Nano-TiO_2_

**Figure 1:**
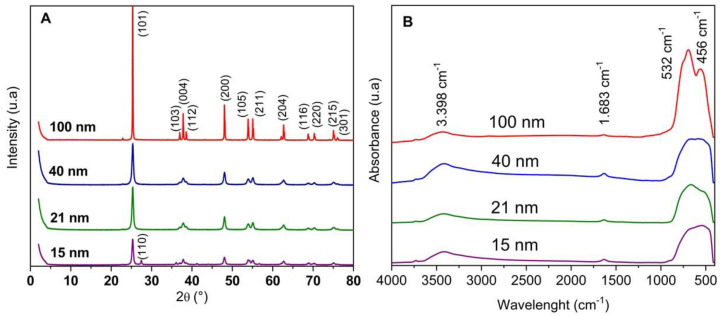
XRD (**A**) and FTIR (**B**) spectra of nano-TiO_2_ with different particle sizes.

X-ray diffractogram analysis allows us to identify the crystalline structures of a chemical compound. Figure 1A shows the diffraction patterns of different samples of nano-TiO_2_. All samples showed the characteristic typical anatase diffraction 2θ peaks at 25.41°, 37.94°, 48.05°, 54.13°, 55.39°, 70.24° and 75.14° which correspond to planes (101), (004), (200), (105), (211), (220) and (215) of its crystalline structure (JCPDS 84-1286) [18]. Anatase has a tetragonal structure of crystallite, and the lattice parameters a, b and c were calculated, considering a = b due its structure. The values were (1) a: 3.7889, 3.7787, 3.7787 and 37824 Å and (2) c: 9.4983, 9.4597, 9.4983 and 9.5047 Å for 15, 21, 40 and 100 nm, respectively. These values are in agreement with those reported for an anatase standard (a = 3.7848 and c = 9.5124 Å) [19].

It should be noted that the peak intensities increased with increasing particle size. This could be observed in nano-TiO_2_ (100 nm), where the peak intensity was higher than for samples of 40, 21 and 15 nm. This was because nano-TiO_2_, with lower particle size is composed of irregular and amorphous structures crystals which produce a reduction of the peak intensities [20]. These amorphous zones revealed a broad pattern of low intensity in the shape of the peaks; however, the effect of the amorphous materials on the broadening of the XRD patterns of the nano-TiO_2_ samples was negligible.

The FTIR spectra of different nano-TiO_2_ samples are shown in Figure 1B. According to this analysis, all samples of nano-TiO_2_ had similar spectra. A small band was observed around 3400 between 3500 and 3000 cm^−^^1^ and there was a small peak that could be assigned to the stretching of surface hydroxyl groups of TiO_2_. In addition, there was a band around 1680 and between 1500 and 1600 cm^−^^1^. This small band could evidence the presence of -OH bonds due to of moisture content in the TiO_2_ [21]. Finally, the broad band located at 750–500 cm^−^^1^ was assigned to the bending vibration of Ti-O-Ti bonds in the TiO_2_ lattice. An important band close to 1000 cm^−^^1^ to 400 cm^−^^1^ was also observed. This band reflected the vibration of O-Ti-O bonds in the molecules [20].

**Figure 2:**
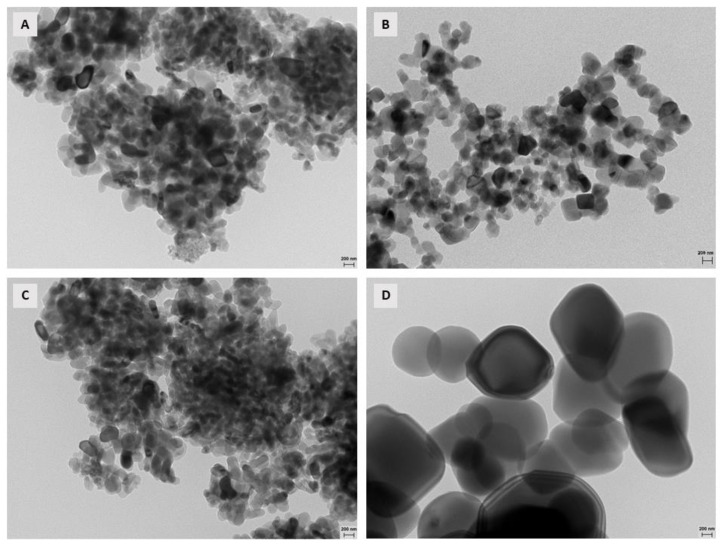
TEM micrographs of the different nano-TiO_2_ samples: 15 nm (**A**), 21 nm (**B**), 40 nm (**C**) and 100 nm (**D**). Magnification 50 Kx.

Figure 2 shows the TEM images of different nano-TiO_2_ samples. The morphology was determined through TEM microscopy. The nanoparticles were characterized by a rounded shape and the formation of agglomerates (confirmed by PS analysis). From this analysis it was possible to determine the sizes and shapes of the nanoparticles. The nano particles in samples were sphere-shaped, while the sizes, determined through DLS equipment, were heterogeneous: 272.2 ± 22.6; 335.5 ± 44.5; 597.4 ± 55.2 and 779.9 ± 28.5 nm for samples of 15, 21, 40 and 100 nm, respectively. These results show that the agglomeration of the nanoparticles overestimated the real particle size, resulting in sizes above those reported in the data sheets. The particle sizes increased due to all samples being agglomerated and was caused by the characteristics of the samples because these were powdered nanoparticles.

As the nanoparticles would be used in the elaboration of nanocomposites through an extrusion process, it was important to determine their thermal stability. For this reason, the nanoparticles were analyzed by TGA. All samples exhibited a single mass loss of about 5% at 100 °C which was associated with the evaporation of water (previously evidenced in the FTIR analysis). At higher temperatures, no changes in nano-TiO_2_ mass were observed, which confirms the high thermal stability of this compound [22]. With respect to thermal properties, these were determined through thermogravimetric analysis (TGA). All samples had a difference in weight between 30 and 100 °C, due to evaporation of water; this could be attributed to a certain humidity present in the samples. Therefore, it can be said that there was a weight loss of approximately 5% for all samples of nano-TiO_2_. The presence of water could be observed through the FTIR test, which is explained above. However, after this initial degradation, the mass of active compound (TiO_2_) was constant until 900 °C, so it is possible to conclude that nano-TiO_2_ is highly temperature-stable, and this characteristic is desirable when producing nanocomposites by the extrusion process. These results were expected since TiO_2_ is an inorganic chemical compound, which is highly stable at high temperatures [22].

### 3.2. Photocatalytic Activity

**Figure 3:**
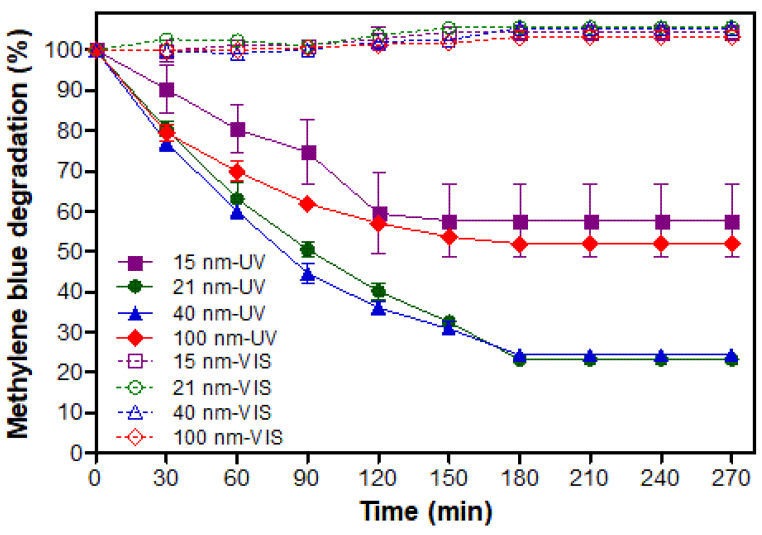
Degradation kinetics of methylene blue under UV and VIS irradiation.

In order to assess the photocatalytic activity of TiO_2_ nanoparticles to oxidize organic molecules, their activity against methylene blue (MeB) was determined. It is well-known that the use of nanoparticles provides a larger contact area during photocatalytic reactions and furthermore that it is favored when anatase structure is used [21]. This assay considered the monitoring of MeB concentration for 270 min when this compound was exposed to both nano-TiO_2_ of different sizes and UV or VIS light (Figure 3). The results showed that the type of light had a clear effect on the degradation of MeB. Thus, samples irradiated with UV light were able to significantly degrade the MeB, while samples irradiated with VIS light were not able to affect this compound. 

This can be explained due to the characteristic of the crystalline structure that was used. It is known that anatase does not have photocatalytic activity under VIS light due to its wide band gap (3.2 eV) [23]. Hence, only UV radiation has sufficient energy to generate reactive oxygen species (ROS), which can act as oxidants against organic molecules [24]. ROS are formed from photoexcitation of electrons (e−) in TiO_2_. The electrons move from the valence band to the conduction band leaving holes (h+) in the first band [7]. Here pairs of e− and h+, called excitons, are generated, causing oxidation/reduction reactions with H_2_O and O_2_ which are present in the reaction medium. In this way, ROS are produced. Within these species, the hydroxyl radicals (▪OH) are recognized as being responsible for the degradation of MeB and other organic compounds into CO_2_ and H_2_O [24]. On the other hand, despite sunlight having a low amount of UV light (~5%), this is not enough to activate the nanoparticles. Consequently, it is essential to use artificial UV light to activate TiO_2_ anatase [25].

Regarding particle size, it was observed that nano-TiO_2_ samples of 21 and 40 nm were more active in degrading MeB than nanoparticles of 15 and 100 nm. These results were expected, especially for the 100 nm particles since it has been reported that the photocatalytic activity of TiO_2_ decreases with increasing particle size due to decreasing surface area [24]. However, in the case of the 15 nm particles the explanation would be different. Once e− and h+ are generated by the interaction between TiO_2_ and UV light, two processes can occur: (i) recombination of e− and h+ and the dissipation of their energy in the form of electromagnetic radiation or heat and (ii) migration to the TiO_2_ surface to react by absorbing molecules [7]. Thus, the e- can move to the particle surface through electron traps on the crystalline net, which can be of two types—superficial or deep. Superficial traps are beneficial to the electron migration process due to the photocatalyser surface, which allows the reaction, whereas the deeper traps constitute recombination centers. Therefore, when particle size reduces under an optimal size in nanocrystalline TiO_2_, most electrons and holes generated close to the surface recombine faster than the interfacial charge transfer processes. Similar results were obtained in a study where different particle sizes (8, 18 and 27 nm) of the crystalline structure of titanium dioxide were evaluated according to their photocatalytic properties against MeB. In this study, the authors were able to evidence that smaller-sized nanoparticles (8 nm) showed lower photocatalytic activity against organic molecules [26]. Additionally, the agglomeration of nanoparticles could be another factor to consider. As observed in the TEM analysis (Section 3.1), agglomeration was more important in smaller particles. Thus, agglomeration would allow the interaction of light only with the most exposed particles in the agglomerate, thus affecting the photocatalytic process.

### 3.3. Evaluation of Ethylene Degradation by Nano-TiO_2_

**Figure 4:**
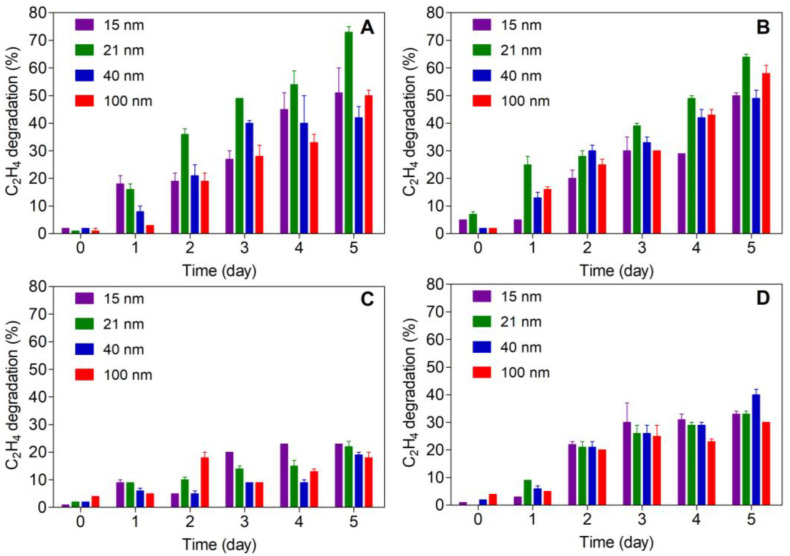
Ethylene degradation of TiO_2_ nanoparticles under different types of light and irradiation times: 5 min—UV (**A**), 15 min—UV (**B**), 5 min—VIS (**C**) and 15 min—VIS (**D**).

The ethylene-removal capacity, which is expressed as the reduction percentage of the ethylene concentration of different nanoparticles, was determined over a 5-day period under two types of light (UV or VIS) and two irradiation times (5 or 15 min) (Figure 4).

In general, as time increased, an increase in ethylene removal was evidenced for all the nanoparticles studied; however, significant differences were observed when comparing the type of light used. Thus, the nanoparticles irradiated with UV light showed higher ethylene-removal capacity than those treated with VIS light (Table 1 and Table 2, respectively). However, there were significant variations in this activity for the different nanoparticle sizes at the two irradiation times. As a result, the 21 nm samples were the most active, reducing the ethylene concentration by about 70% with no significant differences of irradiation time in the activity against the olefin, while the 40 nm particles were the least active with a reduction of about 45%. On the other hand, the samples treated with VIS light were not able to exceed 40% ethylene removal, with 15 nm samples showing the highest activity, while 100 nm samples were the least active. In contrast to the samples treated with UV light, the samples treated with VIS light showed significant differences with increasing light exposure time, which is in agreement with the low content of UV radiation in VIS light. From this analysis, the photocatalytic effect of titanium dioxide on ethylene was confirmed.

On the other hand, and unlike the results observed in Section 3.2, VIS light showed an effect on ethylene. Although this activity was lower than that observed with UV light, some effect was evidenced in the ethylene control. This agrees with a previous study carried out in our group where nano-TiO_2_ was incorporated into a low-density polyethylene (LDPE) film and its effect on banana ripening was evaluated. In this case, a clear effect of non-irradiated films on physiological aspects of the fruit, such as ripening index and color, was observed [27]. As mentioned above, the major ethylene degradation condition is with UV light, because this kind of light has enough energy to overcome the TiO_2_ band energy and cause its photoexcitation, which in turn generates electron–proton pairs (e− *-* h+) that subsequently generate ROS, responsible for oxidizing ethylene to convert it into CO_2_ and H_2_O [15]. Based on these results, it was considered that the best condition for the removal of ethylene by nanocomposites was the 21 nm sample with UV irradiation for 5 min.

### 3.4. Elaboration and Characterization of TiO_2_ Nanocomposites through Extrusion Process

To take advantage of the photocatalytic activity and the ethylene-removal ability observed with 21 nm TiO_2_ particles, these nanoparticles were used to produce nanocomposites in pellet form. The pellets were produced through a microscale extrusion process to determine the possibility of scaling up the materials developed here to the industrial sector for F&V preservation. The pellets were made with different concentrations of TiO_2_ (0, 5 and 10% wt.) in two polymeric matrices (Mater-Bi^®^ (MB) and poly(lactic acid) (PLA)). 

#### 3.4.1. Optical Properties

Material color for food packaging is an important factor and determines the first impression of consumers about a product [28]. In addition, color can indicate the quality of the dispersion of the additives incorporated into a polymer matrix. Nano-TiO_2_ has a white color and good properties of light refraction, so that the visual color-change of the nanocomposite is usually not significant even with the addition of this oxide. However, if the nanocomposite matrix color is different between samples, the color characteristics of added TiO_2_ nanocomposite can vary slightly from one sample to another [15]. To determine if there were differences of color between samples, both for matrix type and nano-TiO_2_ concentration factors, CIELAB parameters were determined (Table 3). 

It can be observed that nano-TiO_2_ had a greater impact in PLA than in MB, especially in the case of parameters a*, b* and ΔE_00_. High values of L for PLA and MB nanocomposites indicate that all samples tended to white. Likewise, in both materials the variations in parameter a* indicate a shift towards red color, while the decrease in parameter b* reflects a shift towards yellow as the oxide content in the nanocomposites increased. Regarding the ΔE_00_ parameter, it can observed that there were significant differences between polymer matrices and nano-TiO_2_ concentrations, however, the change was more important for PLA. As with the other parameters, the white coloration of the MB control and the transparent PLA control would be the cause of the observed changes.

#### 3.4.2. Morphological Properties

**Figure 5:**
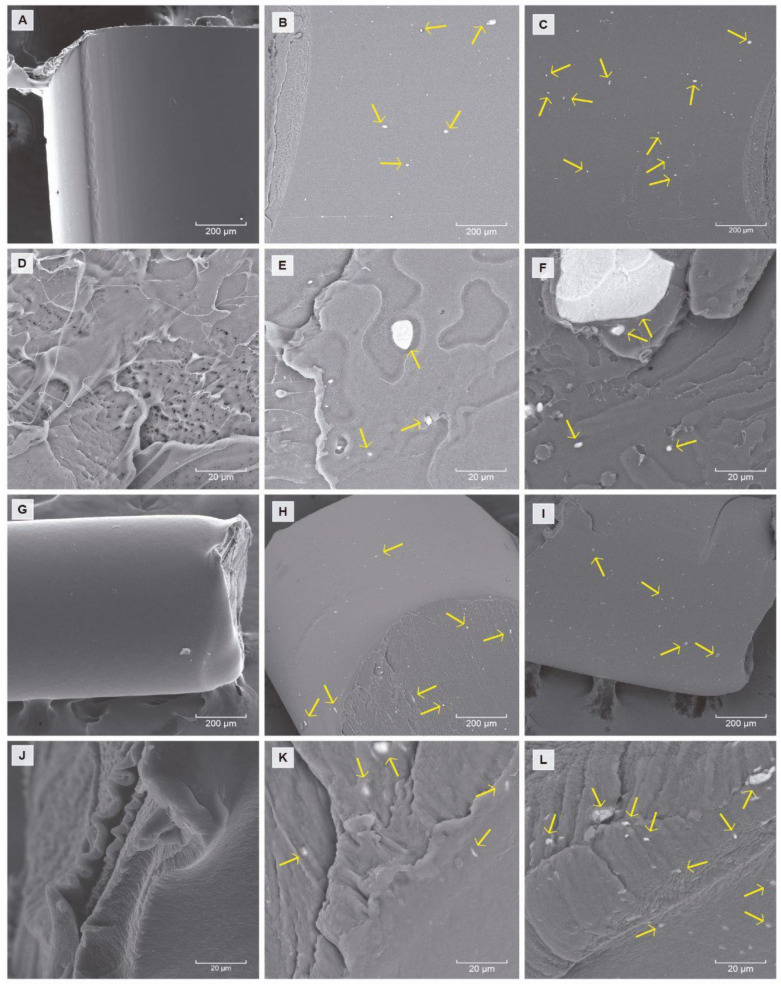
SEM micrographs of nanocomposites: pellet surface of PLA 0%, 5% and 10% (**A**–**C**) and MB 0%, 5% and 10% (**G**–**I**) (200×) and pellet tangential cut of PLA 0%, 5% and 10% (**D**–**F**) and MB 0%, 5% and 10% (**J**–**L**) (1.5 K×). TiO_2_ is marked with yellow arrows.

The dispersion of TiO_2_ into the nanocomposites can play an important role in photocatalytic and ethylene-removal capacity. Figure 5 shows SEM microphotographs of the morphology of different nanocomposites prepared with two polymeric matrixes (PLA and MB) and different nano-TiO_2_ content. The microphotographs show that the presence of nano-TiO_2_ changed the surface of nanocomposites, and there was a strong interfacial adhesion between the two phases. In addition, the formation of agglomerates can be observed, which would be favored with increasing nano-TiO_2_ content.

**Figure 6:**
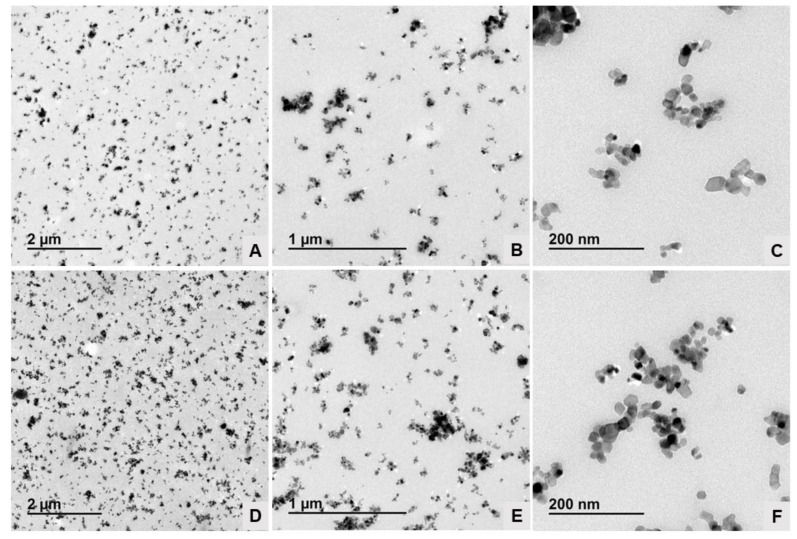
TEM micrographs of PLA nanocomposites: PLA 5% ((**A**–**C**)—1.5, 5.0 and 20 K×) and PLA 10% ((**D**–**F**)—1.5, 5.0 and 20 K×).

Moreover, to examine the dispersion of nano-TiO_2_ into the polymeric matrix, TEM analysis was carried out. Due to the methodology used to prepare the MB samples, they were very flexible and soft and could not be properly embedded in the epoxy resin and only PLA nanocomposites could be analyzed. The results can be observed in Figure 6. In this figure, it is shown that there was an homogeneous distribution of the groups of nanoparticles through the polymeric matrix and it is also possible to observe that there were differences between TiO_2_ concentrations, with PLA 10% having a higher presence of TiO_2_. On the other hand, in microphotographs with a greater magnification (5 and 20 K×) it is possible to see that nano-TiO_2_ nanoparticles tended to agglomerate, as could be seen in Figure 2 for nanoparticle TEM analysis. However, it should be highlighted that in the nanocomposites there were some individual particles dispersed into the PLA matrix, and this behavior did not occur in the TEM assay of nanoparticles; this could be attributed to extrusion process, which is used to disperse, in a better way, the active compound into the polymeric matrix [29]. In fact, the size of the TiO_2_ nanoparticles can be measured from the TEM images at 20 K× and it can be concluded that the TiO_2_ nanoparticles exhibited an average particle average size of 16.0 ± 3.2 nm in PLA nanocomposite with 5% wt. TiO_2_ (Figure 6C), and the average size particle of TiO_2_ in PLA nanocomposite with 10% wt. was 18.2 ± 2.4 nm (Figure 6F), confirming the ability of the extrusion process to disperse such nanoparticles (TiO_2_ < 21 nm particle size).

#### 3.4.3. Evaluation of Ethylene Degradation by PLA and MB Nanocomposites

**Figure 7:**
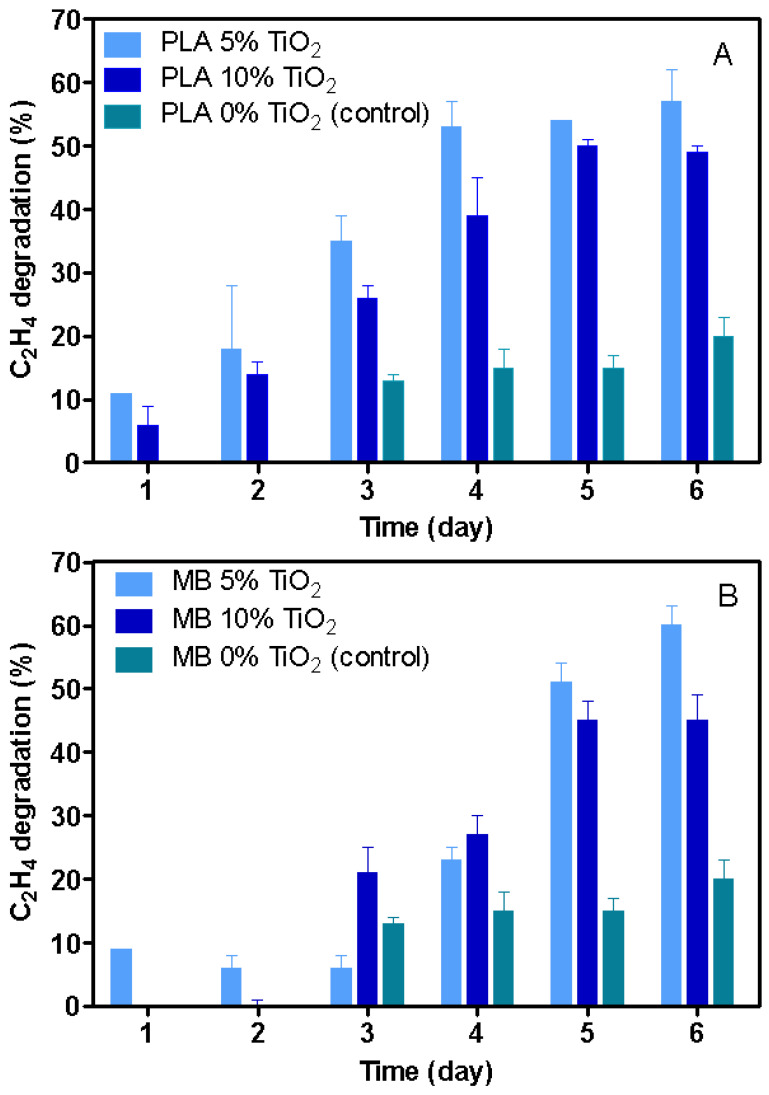
Ethylene degradation of nanocomposites at different TiO_2_ concentrations in PLA (**A**) and MB (**B**).

To determine the ethylene-removal capacity of nanocomposites, the concentration of ethylene was determined over a 6-day period in different samples previously irradiated with UV light (5 min) that were put in contact at 20 °C in hermetically sealed vials. These results can be observed in Figure 7. 

In general, a higher degradation of ethylene was observed as the time increased. Additionally, for both PLA and MB nanocomposites, ethylene degradation was higher for those with 5 wt. % than for those with 10 wt. % of nano-TiO_2_. Thus, ethylene degradation for nanocomposites with 5 wt. % of nano-TiO_2_ was 51 ± 6% for PLA and 57 ± 2% for MB nanocomposites (Table 4). As discussed in the SEM analysis, the nanocomposites with 10 wt. % oxide were characterized by a higher presence of nano-TiO_2_ agglomerates, a situation that generated a less efficient system since it allowed the generation of ROS on the exposed surface and not in the interior of the agglomerate. A similar explanation was given by Shih, Hsin and Lin [30] who found that the photocatalytic degradation of an azo dye from TiO_2_ decreased with larger secondary particle sizes due to the higher intracrystalline diffusional resistance across the agglomerated TiO_2_ particles. Similarly, [3] found a comparable effect of agglomeration of TiO_2_ nanoparticles on their photocatalytic performance in chitosan nanocomposite films, where nanocomposites with a higher concentration of nano-TiO_2_ decreased the ethylene-removal capacity. Likewise, the agglomerate formation was used to explain the low activity of ethylene removers based on zeolite and linear LDPE composites. Although in this case, the removal mechanism was linked to an ethylene sorption on the zeolite. The interaction of the ethylene with the mineral only occurred at the surface level of the agglomerate and not on the zeolite located inside the agglomerate [31].

Moreover, the geometry of the pellets used in this work could also play an important factor in the ethylene-removal capacity. Disregarding Without considering ethylene diffusion through the material, only the nano-TiO_2_ and its agglomerates located on the pellet surface could interact with ethylene to oxidize it. In this respect, changing the extruder configuration to obtain films, and analyzing the distribution of nano-TiO_2_ on the film surface and its effect on ethylene removal will be an important step in this research.

Finally, it is important to mention that the results of this study provide a very good approach to developing potential nanocomposites for fresh food preservation. We showed that it is possible to create nanocomposites with eco-friendly material by an extrusion process, which could be scaled to an industrial level, and that different shapes of active packaging, such as films, could be developed. 

## 4. Conclusions

The analyzed TiO_2_ nanoparticles showed a crystalline structure typical of anatase; moreover, its photocatalytic activity against methylene blue was dependent on the radiation type used (UV or VIS light) and the particle size. In this way, the nano-TiO_2_ 21 nm particle size irradiated under UV light showed the highest photocatalytic activity. Regarding the ethylene removal by the different nanoparticles, the results showed that both types of radiation were able to remove ethylene, however, the nanoparticles activated with UV light presented the highest activity against the gas. In addition, the particle size was also a determinant in ethylene degradation; thus, the 21 nm particle showed the best ethylene removal. On the other hand, it was possible to establish that irradiation time had no effect on the ethylene removal.

Finally, it was possible to elaborate TiO_2_ nanocomposites with PLA and MB through an extrusion process, scalable to the industrial sector. The nanocomposites showed color differences related to the TiO_2_ concentration and the type of polymeric matrix used. In addition, the ethylene-removal study of the nanocomposites showed that all samples were able to reduce the ethylene concentration; however, nanocomposites with 5 wt. % of nano-TiO_2_ had the highest activity, reaching a degradation of 51 ± 6 and 57 ± 2% for PLA and MB nanocomposite, respectively. It can be concluded that the development of TiO_2_ nanocomposites with biopolymers is a good option for the removal of ethylene. This could be an interesting option for fresh-food preservation; however, future studies should consider changing the structure of the nanocomposite, such as into films, to expose a larger amount of nano-TiO_2_ on the surface of the material in order to increase its effectiveness against ethylene.

## Figures and Tables

**Table 1:** Final C_2_H_4_ degradation of different TiO_2_ nanoparticles at different UV irradiation times.

Particle Size (nm)	C_2_H_4_ Degradation (%)	
	Irradiation Time (min)	
	5	15
**15**	52 ± 7 ^ABa^	51 ± 2 ^ABa^
**21**	73 ± 2 ^Ca^	64 ± 1 ^Ca^
**40**	42 ± 4 ^Aa^	49 ± 3 ^Aa^
**100**	50 ± 2 ^Ba^	58 ± 3 ^Ba^

Note: Equal letters mean that there are no statistically significant differences (*p* > 0.05). Upper-case letters: particle-size factor and lower-case letters: irradiation-time factor.

**Table 2:** Final C_2_H_4_ degradation of different TiO_2_ nanoparticles at different VIS irradiation times.

Particle Size (nm)	C_2_H_4_ Degradation (%)	
	Irradiation Time (min)	
	5	15
**15**	23 ± 1 ^ABa^	33 ± 1 ^ABa^
**21**	22 ± 2 ^ABb^	33 ± 1 ^ABa^
**40**	19 ± 1 ^Bb^	40 ± 2 ^Ba^
**100**	18 ± 2 ^Ab^	30 ± 1 ^Aa^

Note: Equal letters mean that there are no statistically significant differences (*p* > 0.05). Upper-case letters: particle-size factor and lower-case letters: irradiation-time factor.

**Table 3:** Colour parameters of PLA and MB nanocomposites with TiO_2_.

Matrix	TiO_2_ Concentration (%)	L*	a*	b*	∆E_00_
PLA	0 (control)	84.8 ± 1.3 ^Ba^	−1.0 ± 0.2 ^Bb^	10.4 ± 0.1 ^Ba^	-
	5	92.8 ± 0.6 ^Ba^	−0.2 ± 0.2 ^Ba^	6.0 ± 0.1 ^Bb^	9.2 ± 0.5 ^Ab^
	10	94.8 ± 1.8 ^Ba^	0.3 ± 0.2 ^Bc^	6.3 ± 0.2 ^Bc^	11.0 ± 1.7 ^Aa^
MB	0 (control)	94.1 ± 0.2 ^Aa^	3.2 ± 0.2 ^Ab^	13.5 ± 0.2 ^Aa^	-
	5	92.1 ± 0.2 ^Aa^	3.0 ± 0.2 ^Aa^	11.5 ± 0.2 ^Ab^	2.9 ± 0.5 ^Bb^
	10	90.5 ± 0.1 ^Aa^	2.1 ± 0.2 ^Ac^	9.8 ± 0.2 ^Ac^	5.3 ± 0.4 ^Ba^

Note: Equal letters mean that there are no statistically significant differences (*p* > 0.05). Upper-case letters: matrix factor and lower-case letters: TiO_2_ concentration.

**Table 4:** Final C_2_H_4_ degradation by different TiO_2_ nanocomposite concentrations.

TiO_2_ Concentration (%)	C_2_H_4_ Degradation (%)	
	Matrix	
	PLA	MB
**0 (control)**	20 ± 2 ^Ac^	18 ± 2 ^Ac^
**5**	51 ± 6 ^Aa^	57 ± 2 ^Aa^
**10**	46 ± 2 ^Ab^	51 ± 5 ^Ab^

Note: Equal letters mean that there are no statistically significant differences (*p* > 0.05). Upper-case letters: matrix factor and lower-case letters: TiO_2_ concentration.

## Data Availability

Not applicable.
